# Preclinical Cancer Chemoprevention Studies Using Animal Model of Inflammation-Associated Colorectal Carcinogenesis

**DOI:** 10.3390/cancers4030673

**Published:** 2012-07-16

**Authors:** Takuji Tanaka

**Affiliations:** 1 Cytopatholgy Division, Tohkai Cytopathology Institute, Cancer Research and Prevention (TCI-CaRP), 5-1-2 Minami-uzura, Gifu 500-8285, Japan; E-Mail: takutt@toukaisaibou.co.jp; Tel.: +81-58-273-4399; Fax: +81-58-273-4392; 2 Department of Tumor Pathology, Gifu University Graduate School of Medicine, 1-1 Yanagido, Gifu 501-1194, Japan

**Keywords:** inflammatory bowel disease, colorectal cancer, chemoprevention, animal model, AOM, DSS, morin, bezafibrate, valproic acid

## Abstract

Inflammation is involved in all stages of carcinogenesis. Inflammatory bowel disease, such as ulcerative colitis and Crohn’s disease is a longstanding inflammatory disease of intestine with increased risk for colorectal cancer (CRC). Several molecular events involved in chronic inflammatory process are reported to contribute to multi-step carcinogenesis of CRC in the inflamed colon. They include over-production of free radicals, reactive oxygen and nitrogen species, up-regulation of inflammatory enzymes in arachidonic acid biosynthesis pathway, up-regulation of certain cytokines, and intestinal immune system dysfunction. In this article, firstly I briefly introduce our experimental animal models where colorectal neoplasms rapidly develop in the inflamed colorectum. Secondary, data on preclinical cancer chemoprevention studies of inflammation-associated colon carcinogenesis by morin, bezafibrate, and valproic acid, using this novel inflammation-related colorectal carcinogenesis model is described.

## Abbreviations

ACF: aberrant crypt foci
AD: adenoma
ADC: adenocarcinoma
AOM: azoxymetheane
5-ASA: 5-aminosalicylic acid
CD: Crohn’s disease
COX: cyclooxygenase
CRC: colorectal cancer
DMH: 1,2-dimethylhydrazine
DSS: dextran sodium sulfate
H&E: hematoxylin and eosin
HATs: histone acetylases
HCC: hepatocellular carcinoma
HDACs: histone decarboxylases
HDACIs: histone decarboxylase inhibitors
HIF: hypoxia-inducible factor
HMG-CoA: 3-hydroxy-3-methylglutary coenzyme A
IBD: inflammatory bowel disease
IL: interleukin
iNOS: inducible nitric oxide synthase
KAD: Kyoto *Apc* Delta
MAM: methylazoxymethanol
NF-κB: nuclear factor-kappaB
NSAIDs: non-steroidal anti-inflammatory drugs
PhIP: 2-amino-1-methyl-6-phenylimidazo[4,5-*b*]pyridine
PPARs: peroxisome proliferator-activated receptors
PSC: primary sclerosing cholangitis
RXR: retinoid X receptor
TNF: tumor necrosis factor
Stat3: signal transducer and activator of transcription
UC: ulcerative colitis
UDCA: ursodeoxycholic acid
VPA: valproic acid

## 1. Introduction

An association between inflammation and cancer has been suggested for a long time [1] and it is now well-recognized that inflammation is involved in carcinogenesis in several tissues [2,3]. Patients with inflammatory bowel disease (IBD), especially major types of IBD ulcerative colitis (UC) and Crohn’s disease (CD) have a significantly increased risk of developing premalignancy (dysplastic lesions) and malignancy (adenocarcinoma, ADC) in the colorectum [4,5,6]. Although UC-associated colorectal cancer (CRC) accounts for only less than 2% of all CRCs in the general population, it is responsible for 10–15% of deaths in the UC patients [7]. The risk of CRC increases in relation to the degrees of inflammation and the disease duration (duration/risk = 10 years/1.6%, 20 years/8.3%, and 30 years/18.4%) in UC patients [8]. Even younger patients with UC have high risk of CRC [9]. CD is also associated with an increased risk of large and small bowel ADC [10]. Patients with CD have an increased cumulative risk for CRC, from 2.9% at 10 years to 8.3% after 30 years of disease [10]. Patients with UC as well as those with CRC have been increasing in Asian countries including Japan, similarly to Western countries [11]. Therefore, it is necessary to investigate the mechanisms of CRC development with the background of inflammation for establishing the countermeasure strategy such as chemoprevention [12,13,14]. Also, a novel animal model is required [15,16,17,18,19], as until now there have been few useful ones.

Along with the development of surveillance colonoscopy or prophylactic colonoscopy, recently the concept of chemoprevention has gained increasing importance [20]. Many natural or synthetic pharmacological agents have been evaluated for their chemopreventive efficacy for UC-associated CRC using animal models. The ideal chemopreventive agents would be effective for preventing neoplastic progression, safe (without or low side-effects), and inexpensive [20,21]. The most frequently used chemopreventive agents in UC patients are 5-aminosalicylic acid (5-ASA) compounds (mesalazine and sulfasalazine) as well as ursodeoxycholic acid (UDCA), which is applied in patients with primary sclerosing cholangitis (PSC) [22] being one of the extra-intestinal manifestations. Although the chemopreventive role for UDCA in PSC patients is generally accepted [23,24], there is still debate regarding the chemopreventive capability of 5-ASA derivatives in the patients without PSC [21]. While there are numerous studies supporting the chemopreventive efficacy of 5-ASA [25,26], several studies [27,28] could not find significant reduction in UC-associated dysplasia and CRC. We have reported that chemopreventive efficacy of UDCA is superior to that of 5-ASA in the mouse azoxymethane (AOM)/dextran sodium sulfate (DSS) model [15,17,18] of colitis-related colorectal carcinogenesis [29]. Therefore, further pharmacological candidates and potential targets should be evaluated for chemoprevention in UC-associated CRC [12,13,14,17,30].

This article describes our short-term mouse and rat CRC models with the background of colitis mimicking human UC and our exploration of chemopreventive agents [12,13,14], and finally summarizes our recent data on chemopreventive abilities of morin, bezafibrate, and valproic acid (VPA) against inflammation-related mouse or rat colorectal carcinogenesis.

## 2. Development of an Inflammation-Associated CRC Model

### 2.1. AOM/DSS Mouse Model

**Figure 1:**
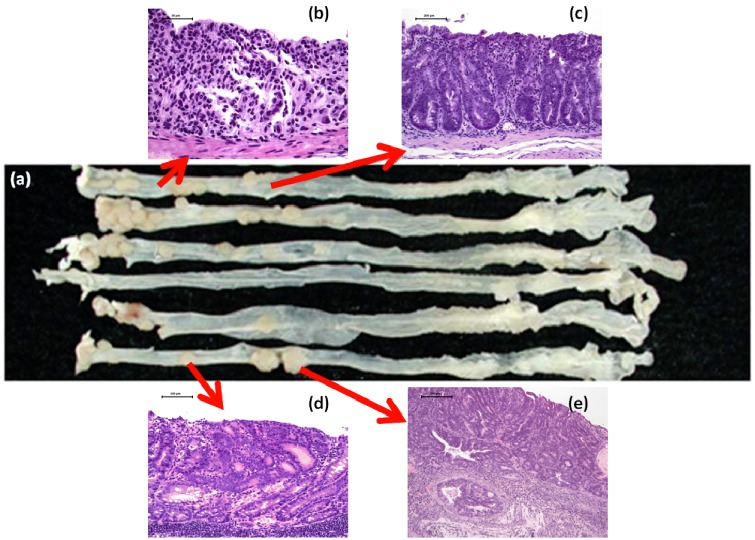
(**a**) Macroscopic view of colorectal tumors developed in mice that received AOM and DSS. Histopathology of (**b**) severe mucosal ulcer; (**c**) dysplastic crypts, (**d**) colonic adenoma (AD), and (**e**) adenocarcinoma (ADC) in a mouse that received AOM and DSS.

Rats have mostly been employed for animal colorectal carcinogenesis models, and AOM, methylazoxymethanol (MAM) acetate, and 1,2-dimethylhydrazine (DMH) have been widely used as colorectal carcinogens [15]. About 30 weeks are required for development of CRC in about half of rats that are initiated with these colonic carcinogens. On the other hand, in experiments and studies using mice, multiple administrations of the colorectal carcinogens are required and it takes a long-term of 40 weeks or longer to develop CRC [31]. Therefore, I have developed a novel mouse model that would produce CRC in a short-term in the inflamed colorectum [18]. AOM-DSS, here called the TANAKA model [15,17,18], is a well-characterized experimental model for UC-associated colorectal carcinogenesis (Figure 1a–e) [18,32,33,34,35,36]. Mice that received a single injection of a low dose of the classic colon carcinogen AOM prior to administration of DSS in drinking water develop inflammation and mucosal ulcer (Figure 1b) [18,37,38] as well as dysplasia (Figure 1c), adenoma (AD) (Figure 1d) and ADC (Figure 1e) with pathologic features that resemble those of human UC-associated neoplasia [18,33,34,36]. The extent of neoplastic lesions depends on several factors like strain susceptibility as well as duration, dosage, and schedule of cyclic DSS application [39,40,41].

To settle the issue of the influence of peroxisome proliferator-activated receptor (PPAR) agonists on colorectal carcinogenesis, which has been a topic since 1998 [42,43,44], we confirmed that colitis induced by DSS, which is a non-genotoxic carcinogen [45,46], using aberrant crypt foci (ACF) as a biomarker [16,47,48,49], had tumor promoter activity to enhance development of ACF in rats and hypothesized that a combination of DSS and AOM would induce CRC in a short-term period in mice as well [50].

### 2.2. DMH/DSS and 2-Amino-1-methyl-6-phenylimidazo[4,5-b]pyridine (PhIP)/DSS Mouse Models

Instead of AOM, experiments with DMH [51] or a heterocyclic amine, PhIP [52] as an initiator (colon carcinogen) and followed by DSS treatment resulted in rapid development of colorectal neoplasms. Histopathologically, ADC induced by DMH/DSS showed severer atypia and more aggressive biological nature than that induced by AOM/DSS. As noticed in the cancers induced by AOM/DSS, the ADC cells developed in the inflamed colon of mice that received DMH and DSS were positive for cyclooxygenase (COX)-2, inducible nitric oxide synthase (iNOS), and β-catenin. Mutation patterns of the *β-catenin* gene slightly differ among the ADCs that were induced by the different treatment regimens: AOM/DSS, codon 32–34, 37, and 41; DMH/DSS, codon 32, 34, 37, and 41; and PhIP/DSS, codon 32 and 34. However, these mutations was limited to the codon 32–34, 37, 41, and 45 that played an important role in degradation of β-catenin protein.

### 2.3. DSS Promotes CRC Development in Apc^Min/+^ Mice

In *Apc^Min/+^* mice, known as an animal model for familial adenomatous polyposis (FAP), multiple tumors (tubular ADs) develop in the small intestine, instead of the large intestine in human FAP, and markedly few tumors develop in the large bowel. However, dysplastic crypts are observed in the colonic mucosa of *Apc^Min/+^* mice [53,54]. Therefore, DSS possibly enhances the growth of dysplastic crypts, and finally the lesions progress to ADCs. To investigate whether DSS-induced inflammation in the colonic mucosa would accelerate the growth of dysplastic crypts, *Apc^Min/+^* mice were given drinking water containing 2% DSS for one week without the initiation (carcinogen) treatment [53]. Surprisingly, multiple colorectal tumors, which were histopathologically tubular ADs and ADCs, developed four weeks after the end of DSS treatment. Immunohistochemistry showed that the developed colorectal ADCs were positive against β-catenin, COX-2, iNOS, and p53 antibodies, suggesting that these factors were involved in the development of colorectal neoplasms in the *Apc^Min/+^* mice by the DSS treatment, in addition to oxidative stress and nitrosative stress. The findings suggested that DSS-induced inflammation in the large bowel of *Apc^Min/+^* mice exert powerful tumor-promotion and/or progression effects on the growth of dysplastic crypts, which had already existed after the birth [53,54].

### 2.4. AOM/DSS and DMH/DSS Rat Models

**Figure 2:**
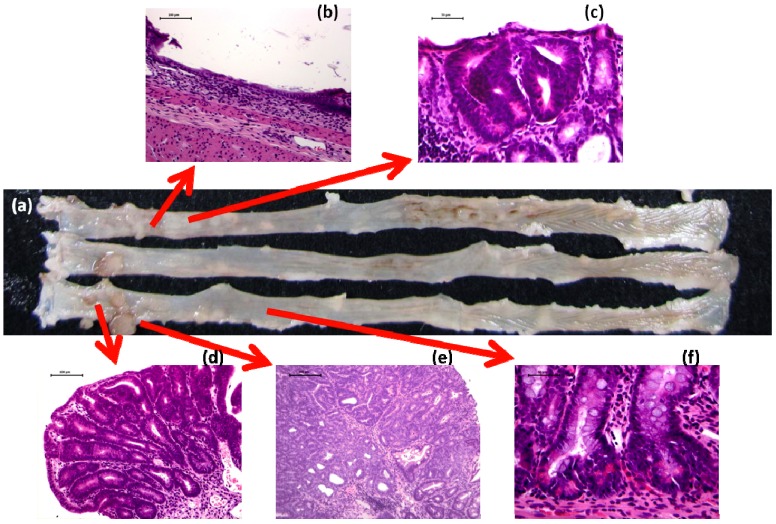
(**a**) Macroscopic view of colorectal tumors developed in KAD rats that received AOM and DSS. Histopathology of (**b**) severe mucosal ulcer; (**c**) dysplastic crypts; (**d**) colonic adenoma (AD); (**e**) adenocarcinoma (ADC), and (**f**) cryptal cells containing Paneth’s granules in a mouse that received AOM and DSS.

The mouse inflammation-associated colorectal carcinogenesis model was named the TANAKA model. With this model it was possible to induce colorectal tumors in a short-term period in rats as well as by similar treatment regimens (AOM/DSS and DMH/DSS) [55,56]. The administration dose of colon carcinogens for initiation is too low to induce colon tumors, but it can initiate or induce DNA modification [18]. Treatment with DSS followed by AOM did not produce colonic neoplasms [18]. The TANAKA model will help advance the research on elucidation of the mechanisms of inflammation-associated colorectal carcinogenesis, inhibition of carcinogenesis, and clarification of the mechanisms of the tumor-promotion ability of DSS. In particular, development of challenging research using the Kyoto *Apc *Delta (KAD) rats (Figure 2a–e) [57] and *gpt *delta rats [58] will give new insight in the pathogenesis of CRC development in the inflamed colon [57]. Interestingly, atypical and neoplastic cells of dysplastic crypts, AD, and ADC in the KAD rats that received AOM and DSS contain Paneth’s granules (Figure 2f), which consist of several anti-microbial compounds and other compounds that are known to be important in immunity and host-defense, in their cytoplasm. We have thus confirmed that in rats with or without genetically alterations colonic tumors are rapidly produced as observed in mice and the rat AOM/DSS and DMH/DSS models can be applied to determine the chemopreventive ability of target compounds.

### 2.5. Detection of Initiators and Promoters in Colorectal Carcinogenesis

We could identify initiators and promoters of colorectal carcinogenesis by modifying the regimens of AOM/DSS and DMH/DSS. When applied test compounds to the initiation treatment instead of AOM or DMH and followed by DSS treatment, we could evaluate initiation activity of test compounds [59]. When test chemicals are applied after initiation treatment with AOM or DMH, we could determine tumor promotion activity of test compounds (unpublished data and [60]). Thus, modification of two regimens in the TANAKA model will determine environmental carcinogens and tumor promoters in the colorectum.

## 3. Exploration of Chemopreventive Agents Using an Inflammation-Associated Colorectal Carcinogenic Model and Elucidation of the Mechanisms

Studies on chemoprevention of inflammation-associated colorectal carcinogenesis by several natural and synthetic compounds against have been reported using the AOM/DSS-induced mouse and rat colorectal carcinogenesis models. Several are promising compounds and their clinical application is expected. Representative compounds are: auraptene and nobiletin from citrus fruits [61], collinin [61], β-cyclodextrin inclusion compounds of auraptene and 4'-geranyloxyferulic acid [62], tricin [35], melatonin [55], urosodeoxycholic acid [29], COX-2 selective inhibitor nimesulide [63], iNOS selective inhibitors [64], PPAR ligands (troglitazone and bezafibrate) [63], and the lipophilic statin pitavastatin [65]. All these compounds have anti-inflammatory activity and are able to suppress the expression of COX-2, iNOS, and inflammatory cytokines.

## 4. Preclinical *in Vivo* Chemoprevention Studies

Using our animal models of inflammation-associated colorectal carcinogenesis, cancer chemopreventive abilities of candidate compounds, morin, bezafibrate, and VPA in mice or rats were investigated. All animal experiments were performed in accordance with protocols approved by the Animal Care and Use Committee of the Institute, TCI-CaRP.

### 4.1. Morin Study

A flavonol, morin (3,5,7,2',4'-pentahydroxyflavone) found in almonds, mill, fig, mulberry, and other Moraceae, acts as a potent antioxidant, inhibitor of xanthine oxidase, protein kinase C and proliferation, apoptosis inducer and modulator of lipoxygenase and cyclooxygenase activities. This flavone has been reported to inhibit the growth of COLO205 cells in nude mice [66], exhibit intestinal anti-inflammatory activity in the acute phase of rat colitis induced by trinitrobenzenesulfonic acid [67,68]. We have previously reported that morin inhibits AOM-induced putative precursor lesions, ACF, in rats [69] and inhibit chemically-induced rat tongue carcinogenesis [70]. Morin exerts anti-inflammatory effects on septic shock induced by lipopolysaccharide [71]. This study aimed to determine possible inhibitory potential of morin in colitis-associated colon carcinogenesis initiated with AOM and promoted by DSS in male F344 rats.

Materials and methods: A total of 66 male rats (5-week-old) were initiated with a single s.c. injection of AOM (20 mg/kg bw), and then they were given promotion stimuli by the treatment with 1.5% DSS in their drinking water for seven days. They were then given a basal diet containing 50, 250 and 1,000 ppm of morin for 17 weeks. Experimental groups included the AOM/1.5% DSS (n = 14), AOM/1.5% DSS/50 ppm morin (n = 10), AOM/1.5% DSS/250 ppm morin (n = 11), AOM/1.5% DSS/1,000 ppm morin (n = 11), AOM alone (n = 5), 1.5% DSS alone (n = 5), 500 ppm morin alone (n = 5), and untreated (n = 5) groups. At the end (week 20) of the study histopathological analysis of colorectum was performed on hematoxylin and eosin (H&E)-stained histologic sections (3 μm thickness). Proliferation activity of colonic ADCs was determined by immunofluorescence technique using anti-Mcm2 antibody (BD Biosciences PharMingen, Tokyo, Japan). Apoptotic cells were detected by fluorescein in situ tunnel method, TACS TdT kit (R&D Systems, Inc., Minneapolis, MN, USA). Polyamine levels [72] and mRNA expression of nuclear factor-kappaB (NF-κB), tumor necrosis factor (TNF)-α, interleukin (IL)-1β, Stat3, and hypoxia-inducible factor (HIF)-1α [73] in colonic mucosa were determined in mice randomly selected from each group. All measurements were statistically analyzed using either the Tukey multiple comparison post test or Fisher’s extract probability test. Differences were considered to be statistically significant at *p* < 0.05.

**Figure 3:**
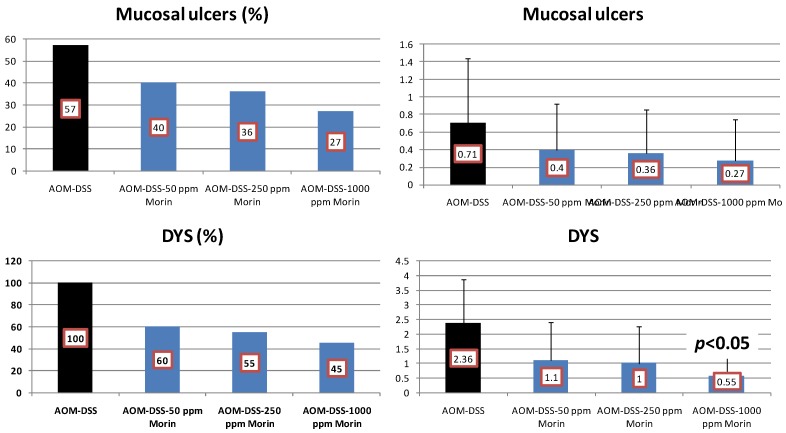
Incidences and multiplicities of mucosal ulcer and dysplastic crypts in the morin study.

**Figure 4:**
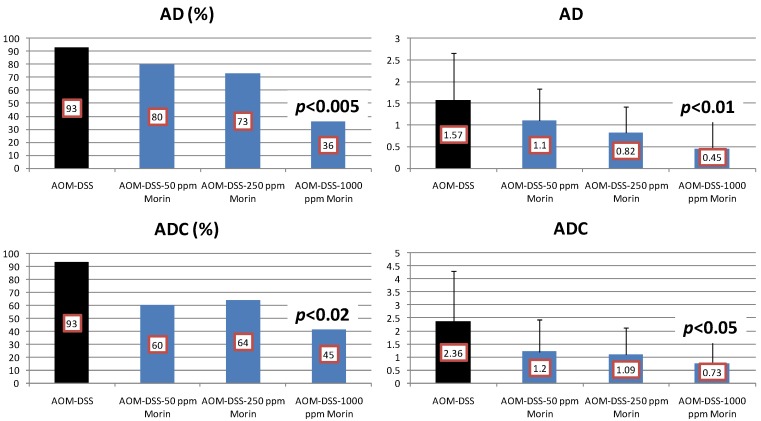
Incidences and multiplicities of adenoma (AD) and adenocarcinoma (ADC) in the morin study.

Results: At week 20, the treatment with morin inhibited colonic mucosal ulcer (Figure 3) and dysplastic crypts (*p* < 0.05 at 1,000 ppm, Figure 3). The incidence (*p* < 0.005) and multiplicity (*p* < 0.01) of colonic AD were significantly reduced by feeding with 1000 ppm morin (Figure 4). Also, dietary administration with 1,000 ppm morin significantly inhibited the incidence (*p* < 0.02) and multiplicity (*p* < 0.05) of colonic ADC (Figure 4), when compared to the AOM/DSS group (93% incidence and 2.36 ± 1.95 multiplicity). Feeding with 50 ppm and 250 ppm morin lowered the incidence and multiplicity of ADC, but the inhibition rates did not reach statistically significance. The treatments also modulated proliferation and apoptosis in ADCs. Mcm2 positive rates (%) of ADCs in rats fed the diets containing 50 ppm morin (n = 6, 78.7 ± 6.8), 250 ppm morin (n = 7, 63.4 ± 6.3, *p* < 0.001), and 1,000 ppm morin (n = 5, 55.6 ± 12.5, *p* < 0.001) were lower than that of rats given AOM and DSS (n = 13, 83.4 ± 8.8). When compared with the AOM and DSS group (n = 13, 8.23 ± 1.24), apoptotic index (%) of ADCs was increased by feeding with morin: 50 ppm morin (n = 6, 10.83 ± 3.66), 250 ppm morin (n = 7, 11.29 ± 3.95), and 1,000 ppm morin (n = 5, 13.00 ± 2.74, *p* < 0.05).

Growth inhibition and apoptosis induction by morin in CRC might be caused by activation of caspase 3 and increase of p21 protein [66]. Suppression of NF-κB-regulated gene products and enhancement of apoptosis induced by TNF [74] also contribute to inhibition of colitis-related colorectal carcinogenesis by morin. Our findings suggest that dietary morin is able to inhibit colitis-related colon carcinogenesis in rats and a flavonol morin is one of the candidates for clinical application of chemoprevention against CRC development in patients with ulcerative colitis.

Because morin can inhibit inflammation, inhibit tumor promotion, suppress tumor growth, and down-regulate the expression of certain genes regulated by NF-κB, it may be possible that morin modulates the activation of NF-κB and NF-κB-regulated gene expression induced by carcinogens, inflammatory agents, and immune modulators. In fact, Manna *et al*. [74] have recently reported morin suppresses the activation of NF-κB and NF-κB-regulated gene expression that leads to enhancement of apoptosis.

### 4.2. Bezafibrate Study

CRC is one of the leading forms of malignancy in the developed countries. Epidemiologic and animal studies have suggested that risk factors for coronary artery disease like insulin resistance and dyslipidemia are probably related to the development of colon cancer [75,76,77]. In particular, nuclear peroxisome proliferator-activated receptors (PPARs), mainly PPARα and PPARγ, which play a central role in lipid and glucose metabolism, had been hypothesized as being involved in colon carcinogenesis [50,78,79]. Furthermore, synthetic PPAR ligands (glitazones and bezafibrate) with proven beneficial effects on insulin resistance and triglyceride levels had been proposed to be candidates as tumor preventive agents [50,63,79]. This study aimed to determine inhibitory potential of bezafibrate in colitis-associated colon carcinogenesis initiated with AOM and promoted by DSS in mice.

Materials and methods: A total of 100 male ICR mice (5-week old) initiated with a single s.c. injection of AOM (10 mg/kg bw) were promoted by 1.5% DSS in their drinking water for seven days. They were then given a basal diet containing 50, 100 and 500 ppm of bezafibrate for 17 weeks. Mice were divided into 8 groups: AOM/1.5% DSS (n = 20), AOM/1.5% DSS/50 ppm bezafibrate (n = 20), AOM/1.5% DSS/100 ppm bezafibrate (n = 20), AOM/1.5% DSS/500 ppm bezafibrate (n = 20), AOM alone (n = 5), 1.5% DSS alone (n = 5), 500 ppm bezafibrate alone (n = 5), and untreated (n = 5) groups. At the end (week 20) of the study histopathological analysis of colorectum was performed on H&E-stained histological sections of 3 μm in thickness. Immunofluorescence technique using anti-Mcm2 antibody (BD Biosciences PharMingen) and fluorescein in situ tunnel method, TACS TdT kit (R&D Systems, Inc.) were used for determination of proliferation activity and apoptosis index of colonic ADCs, respectively. Polyamine levels [72] and mRNA expression of NF-κB, TNF-α, IL-1β, Stat3, and HIF-1α [73] in colonic mucosa were assayed in some mice of each group. Measurements were statistically analyzed using either the Tukey multiple comparison post-test or Fisher’s extract probability test. Differences were considered to be statistically significant at *p* < 0.05.

**Figure 5:**
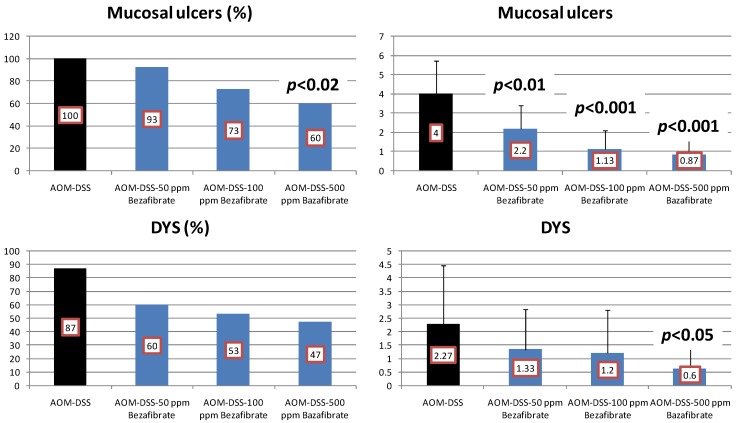
Incidences and multiplicities of mucosal ulcer and dysplastic crypts in the bezafibrate study.

**Figure 6:**
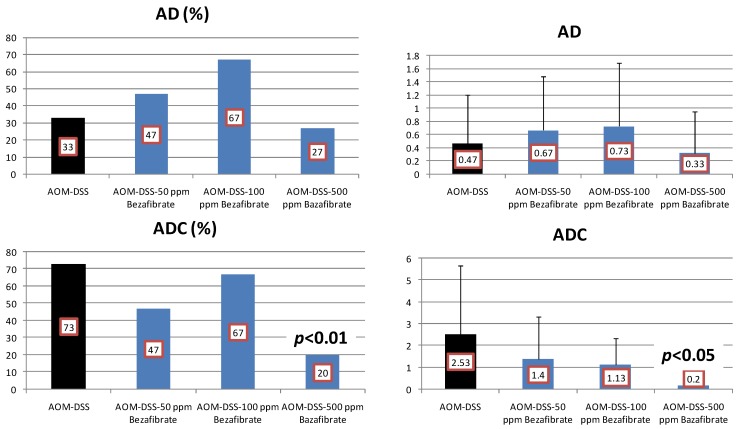
Incidences and multiplicities of adenoma (AD) and adenocarcinoma (ADC) in the bezafibrate study.

Results: At week 20, the bezafibrate feeding inhibited the occurrence of mucosal ulcer (the incidence at 500 ppm, *p *< 0.02; and the multiplicity at 50, 100 and 500 ppm, *p* < 0.01 or *p* < 0.001, Figure 5) and dysplastic crypts (the multiplicity at 500 ppm, *p* < 0.05, Figure 5). As illustrated in Figure 6, he development of colonic ADC was significantly inhibited by feeding with 500 ppm bezafibrate (incidence: 73% reduction, *p* < 0.01; and multiplicity: 92%, *p* < 0.05), when compared to the AOM/DSS group (73% incidence and 2.53 ± 3.14 multiplicity). Feeding with 50 ppm (47% incidence with a multiplicity of 1.40 ± 1.92) and 100 ppm bezafibrate (67% incidence with a multiplicity of 1.13 ± 1.19) also lowered the incidence and multiplicity of ADC, but the inhibition was not statistically significant when compared to the AOM/DSS group (Figure 6). Although statistically insignificant, feeding with 50 and 100 ppm bezafibrate increased the incidence and multiplicity of ADs, but 500 ppm bezafibrate decreased the development of ADs (Figure 6). These findings may suggest the threshold of chemopreventive ability of bezafibrate. Feeding with bezafibrate lowered Mcm2 positive rates (%) of ADCs, when compared with the AOM and DSS group (n = 10, 72.9 ± 10.1): 50 ppm bezafibrate (n = 7, 54.3 ± 9.5), 100 ppm bezafibrate (n = 4, 38.8 ± 7.4, *p* < 0.01), and 500 ppm bezafibrate (n = 3, 22.7 ± 3.2, *p* < 0.001) were lower than that of rats given AOM and DSS (n = 13, 83.4 ± 8.8). When compared with the AOM and DSS group (n = 10, 8.18 ± 1.17), apoptotic index (%) of ADCs was increased by feeding with bezafibrate: 50 ppm bezafibrate (n = 7, 10.71 ± 3.35), 100 ppm bezafibrate (n = 4, 13.20 ± 2.49, *p* < 0.05), and 500 ppm bezafibrate (n = 3, 13.25 ± 3.10, *p* < 0.05).

Anti-angiogenic effects and anti-inflammatory activity of PPARα agonist [80] are considered to contribute to inhibition of CRC growth. Down-regulation of the anti-apoptotic gene *Mcl-1* by PPARα agonist [81] causes apoptosis. The findings suggest that dietary bezafibrate is able to inhibit colitis-related colon carcinogenesis in mice and a hypolipidemic drug bezafibrate is one of the candidates for clinical application of chemoprevention against CRC development in patients with ulcerative colitis.

Accumulating animal experimental, human laboratory and epidemiologic data [50,63,79,82,83] support the hypothesis linking triglyceride levels and insulin resistance to the development of colon cancer. These facts emphasize the potential for this cancer to become a preventable disease not only via screening and removal of polyps but through relevant lifestyle changes and pharmacological interventions which can provide even more avenues for prevention [83,84,85].

The incidence of insulin resistance has been increasing in the Western world where colon cancer is the second leading cause of cancer death. This suggests the interrelationship of these conditions. The biological role of PPARs in various diseases, including inflammation and cancer, has been highlighted recently [50,63,78,85,86,87]. PPARs are members of the nuclear hormone receptor family of ligand-activated transcription factors that play a prominent role in the regulation of many metabolic processes. The PPAR isoformes α and γ are important regulators in lipid and glucose metabolism, cell differentiation and inflammatory response [87,88]. These data propose that PPAR may be associated with many aspects of colon cancer development including insulin- and inflammation-related mechanisms. The fibric acid derivative bezafibrate is the pan PPAR (α, β/δ, and γ) activator with predominantly PPARα (as all fibrates) and β/δ effects but also with perceptible PPARγ properties [88,89,90]. The use of bezafibrate is associated with triglyceride-lowering and high density-cholesterol raising effects resulting in decreased systemic availability of fatty acid, diminished of fatty acid uptake by muscle and improvement of insulin sensitization [91,92]. These direct and indirect effects may have contributed to the suppression of the development of colonic tumors in rodents by bezafibrate [50,63,79]. Recently, Tenenbaum *et al*. [93] have reported possible preventive effects of bezafibrate on the development of CRC from patients with coronary artery disease.

### 4.3. Valproic Acid (VPA) Study

Epigenetic modification plays an important role in tumorigenesis. Affecting epigenetic and tumorigenic alterations is a promising strategy for anticancer targeted therapy [94,95,96]. Among the key chromatin modifying enzymes which influence gene expression, histone acetyltransferases (HATs) and histone deacetylases (HDACs) have attracted interest because of their impact on tumor development and progression. Histone deacetylase inhibitors (HDACIs) represent a new and promising class of antitumor drugs that influence gene expression by enhancing acetylation of histones in specific chromatin domains. HDACIs also exert potent anticancer activities inducing cell cycle arrest and apoptosis. Moreover, HDACIs down-regulate genes involved in tumor progression, invasion and angiogenesis. Based on the ability of HDACIs to regulate many signaling pathways, co-treatment of these compounds that are currently under clinical investigation with molecular targeted drugs is a promising strategy against many types of tumors.

VPA (2-propylpentanoic acid) [97] is a well-established drug for the therapy of epilepsy. It is teratogenic when administered during early pregnancy and can induce birth defects such as neural tube closure defects and other malformations. This well-tolerated antiepileptic drug was found to be a powerful HDAC-1 inhibitor [97]. VPA induces differentiation of carcinoma cells, transformed hematopoietic progenitor cells and leukemic blasts from acute myeloid leukemia patients [98]. Our microarray analysis during the AOM-DSS carcinogenesis revealed alteration of Wif-1 expression [34]. VPA has been reported to modify the Wif-1 expression [99]. These findings may suggest possible modifying effects of VPA on AOM-DSS colorectal carcinogenesis. In this study, we determined whether VPA is able to inhibit colitis-associated colon carcinogenesis in mice.

Materials and methods: A total of 85 mice aged five weeks was used and they were divided into 8 groups: AOM/2% DSS (n = 19), AOM/2% DSS/50 ppm VPA (n = 15), AOM/2% DSS/250 ppm VPA (n = 15), AOM/2% DSS/1,000 ppm VPA (n = 16), AOM alone (n = 5), 2% DSS alone (n = 5), 1,000 ppm VPA alone (n = 5), and untreated (n = 5) groups. Mice were initiated with a single s.c. injection of AOM (10 mg/kg bw) were promoted by 2% DSS in their drinking water for seven days. They were then given a basal diet containing 50, 250 or 1,000 ppm of VPA for 17 weeks. At the end (week 20) of the study histopathological examination of large bowel was performed on H&E-stained histological sections (3 μm in thickness). Immunofluorescence technique using anti-Mcm2 antibody (BD Biosciences PharMingen) for evaluating proliferating activity and fluorescein in situ tunnel method, TACS TdT kit (R&D Systems, Inc.) for detecting apoptosis cells were applied on histological sections of colonic ADCs. Polyamine levels [72] and mRNA expression of NF-κB, TNF-α, IL-1β, Stat3, and HIF-1α [73] in colonic mucosa were assayed in some mice of each group.At the end of the study (week 20), Measurements were statistically analyzed using either the Tukey multiple comparison post test or Fisher’s extract probability test. Differences were considered to be statistically significant at *p* < 0.05.

**Figure 7:**
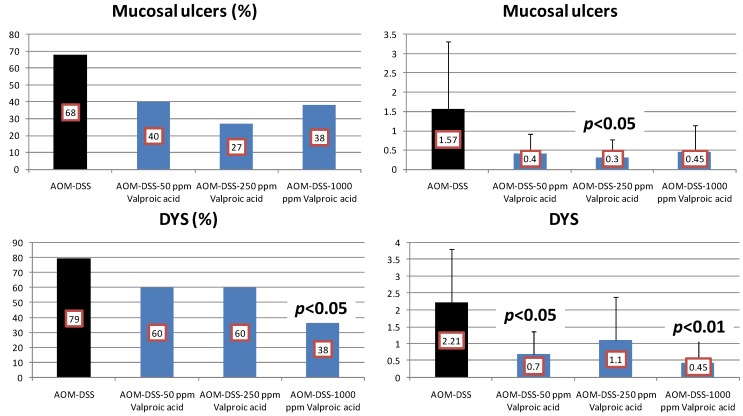
Incidences and multiplicities of mucosal ulcer and dysplastic crypts in the Valproic Acid (VPA) Study.

Results: VPA feeding inhibited the development of mucosal ulcer (Figure 7) and dysplastic crypts (the incidence at 1,000 ppm VPA, *p* < 0.05; and the multiplicity at 50 ppm, *p* < 0.05 and at 1,000 ppm, *p* < 0.01, Figure 7).

**Figure 8:**
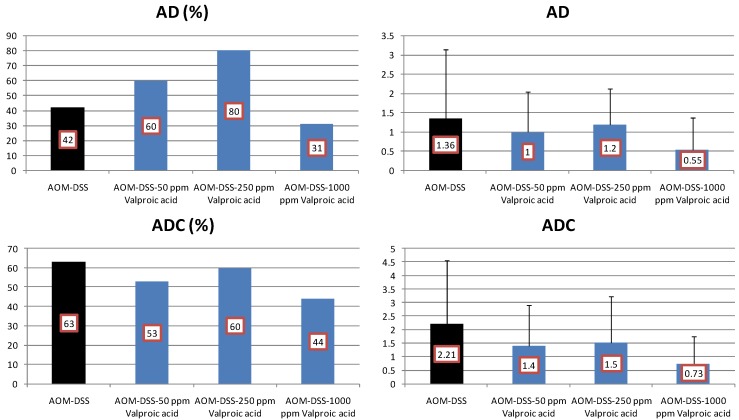
Incidences and multiplicities of adenoma (AD) and adenocarcinoma (ADC) in the Valproic Acid (VPA) Study.

The development of colonic AD and ADC was lowered by feeding with VPA, but the reduction rates were statistically insignificant (Figure 8). When fed with VPA-containing diet, Mcm2 positive rates (%) of ADCs were lower than that of the AOM and DSS group (n = 9, 80.1 ± 6.8): 125 ppm VPA (n = 7, 73.7 ± 8.0), 250 ppm VPA (n = 6, 64.2 ± 2.5, *p* < 0.01), and 1,000 ppm VPA (n = 5, 61.6 ± 9.6, *p* < 0.001). As to apoptotic index (%) of ADCs, the values of mice fed with 125 ppm VPA (n = 7, 10.67 ± 3.44), 250 ppm VPA (n = 6, 12.33 ± 1.75, *p* < 0.05), and 1,000 ppm VPA (n = 5, 12.80 ± 2.39, *p* < 0.05) were higher thab the AOM and DSS group (n = 9, 8.44 ± 1.33).

Our findings suggest slight chemopreventive effects of VPA on colitis-related colon carcinogenesis in mice, suggesting that single use of a HDAC inhibitor VPA is not practical for inhibiting CRC development in inflamed colon. VPA combined with other known chemopreventive or chemotherapeutic agent(s) may exert to inhibit CRC that develop in colitic mucosa.

VPA was studied in combination with all-*trans* retinoid acid in patients with acute myeloid leukemia who were not candidates for intensive chemotherapy [100]. Using human hepatocellular carcinoma (HCC) cells, HepG2, combination treatment with acyclic retinoid and VPA is demonstrated to be an effective regimen for the chemoprevention and chemotherapy of HCC [101]. Acyclic retinoid and VPA cooperatively increase the expression of retinoid X receptor (RXR)-β and p21 (CIP1), while inhibiting the phosphorylation of RXRα, and these effects were associated with induction of apoptosis and the inhibition of cell growth in HepG2 cells. Several HDACIs seem to exert an antitumor effect in a synergistic manner with different anticancer compounds and to overcome the resistance induced by conventional chemotherapeutic drugs [102,103,104,105]. A phase I trial of single agent VPA was reported in patients with newly diagnosed cervical cancer [106]. Twelve patients were included. VPA doses ranged from 20 mg/kg to 40 mg/kg daily for five days. Tumor HDAC activity decreased in 8 patients. Many lines of evidence suggest that tumor cells are characterized by histone hypoacetylation and that over-expression of HDACs is involved in tumorigenesis of various human malignancies [107,108]. Recently, a population-based case-control study with long-term users of VPA has not supported HDAC inhibition by VPA as a pharmacologic principle for general chemoprevention [109]. However, VPA doses (0.35–0.70 mmol/L) used in clinical practice could be too low to achieve cancer preventive effects in contrast the doses (0.50–3.0 mmol/L) that inhibit HDAC. Since VPA is reported to suppress progression of urological malignancies [110,111], it is worthy to evaluate the modifying effects of VPA on different stage of carcinogenesis.

## 5. Effects of Morin, Bezafibrate and VPA on Expression of Pro-Inflammatory Cytokines and HIF-1α and Content of Tissue Polyamines in the Inflamed Colon

IBD represents a dysregulated mucosal immune response to antigens derived from the commensal microbiota in a genetically susceptible host that initially derives from innate immune abnormalities leading to an excessive pro-inflammatory cytokines (T-helper 1, T-helper 2, and T-helper 17 cytokines) derived from CD4^+^ T cells [9,112]. A key point in understanding IBD pathophysiology is to understand the immunoregulatory pathways associated with the intestinal immune system as they apply to IBD. Therefore, in addition to immunotherapy, pro-inflammatory cytokines secreted by innate and adaptive immune cells are targets for IBD treatment [73]. Similarly, cytokines, including NF-κB [73,113], TNF-α [9,35,64], and interleukin (IL)-1β [64,114,115] are potentially molecular targets for inflammation-associated CRC [14]. Potential cancer chemopreventive agents also modulate expression of signal transducer and activator of transcription (Stat3) [55,114], HIF-1α [116,117], and survivin [73] in the target tissues.

Polyamines are organic cations that control gene expression at the transcriptional, posttranscriptional, and translational levels. Multiple cellular carcinogenesis pathways are involved in regulation of transcription and translation of polyamine-metabolizing enzymes [118]. We have reported he importance of research utilizing pharmaceutical inhibitors and cancer chemoprevention against CRC targeting the polyamine pathway [72,119,120].

**Figure 9:**
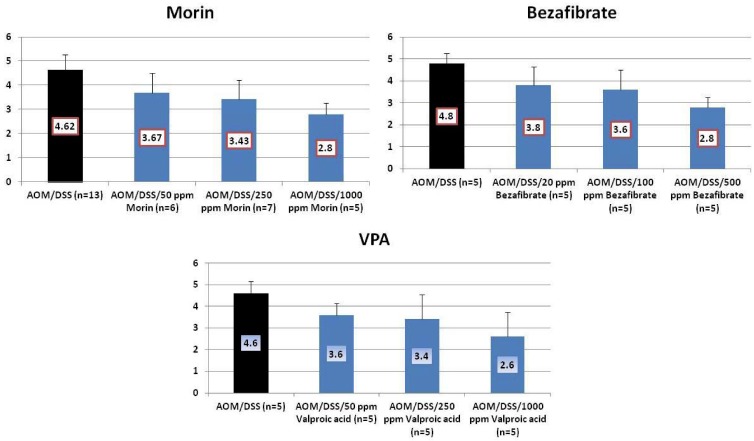
mRNA expression of NF-κB in the colorectum of mice or rats from the morin, bezafibrate, and VPA studies.

**Figure 10:**
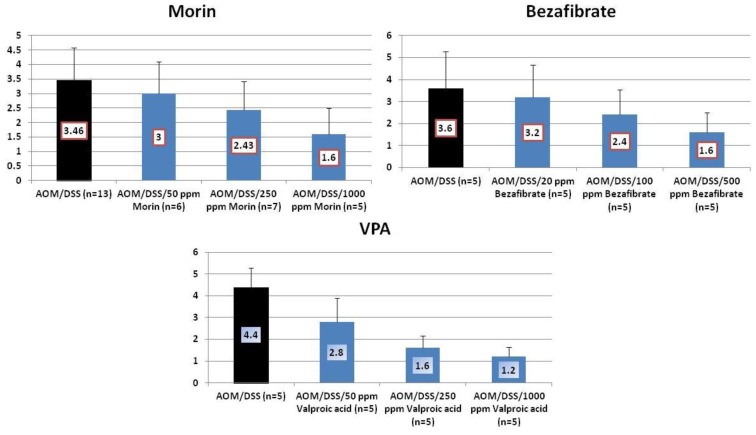
mRNA expression of TNF-α in the colorectum of mice or rats from the morin, bezafibrate, and VPA studies.

**Figure 11:**
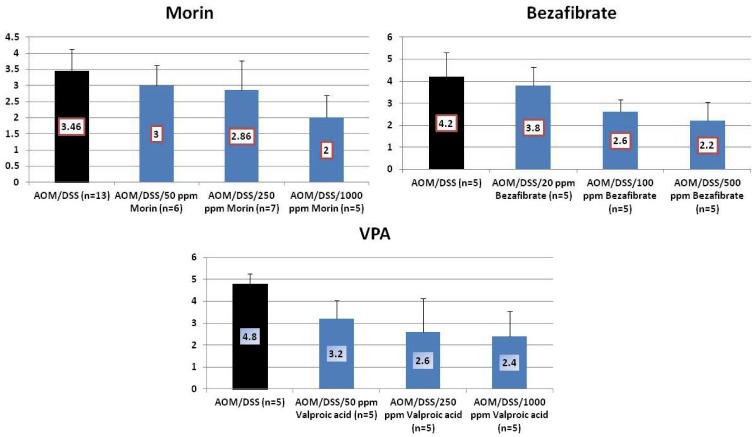
mRNA expression of IL-1β in the colorectum of mice or rats from the morin, bezafibrate, and VPA studies.

**Figure 12:**
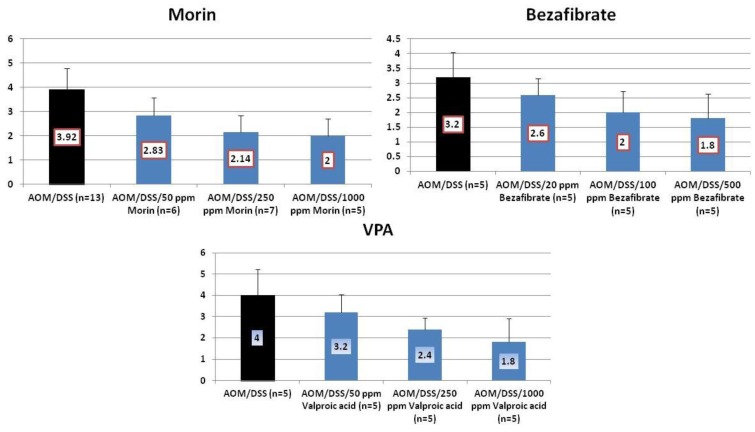
mRNA expression of Stat3 in the colorectum of mice or rats from the morin, bezafibrate, and VPA studies.

**Figure 13:**
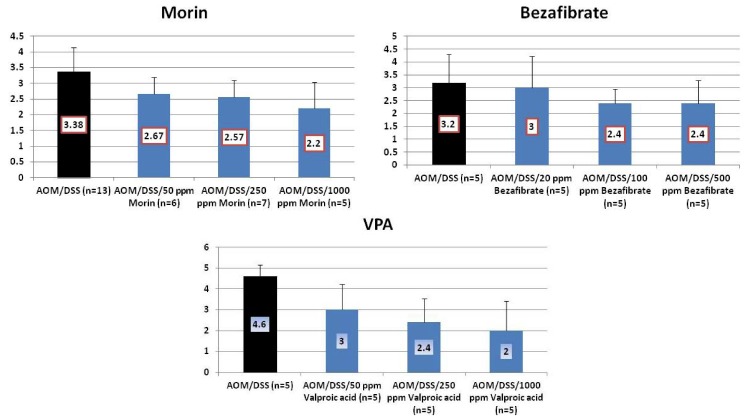
mRNA expression of HIF-1α in the colorectum of mice or rats from the morin, bezafibrate, and VPA studies.

The findings in *in vivo* studies using the TANAKA models suggest that the order of chemopreventive potential of test compounds was bezafibrate > morin > VPA by estimating inhibition rate of CRC development. To investigate the effects of these chemicals on the molecules that are involved in carcinogenesis, we determined mRNA expression of the NF-κB (Figure 9), TNF-α, (Figure 10), IL-1β (Figure 11). Stat 3 (Figure 12), HIF-1α (Figure 13) in colorectal mucosa of mice or rats from the experiments with morin, bezafibrate, and VPA. At sacrifice, each colon was cut open longitudinally, and was flushed clean with PBS. Five animals of each group from three experiments were used for real-time quantitative RT-PCR analysis. Their distal colon was taken for total RNA isolation. For total RNA isolation, epithelial cells were scraped from the underlying muscle layer with a glass microscope slide, were homogenized on ice in lysis buffer (Qiagen, Tokyo, Japan), and were frozen at −80°C until RNA was isolated. Total RNA was extracted from colonic mucosa using the RNeasy Mini Kit (Qiagen) according to the manufacturer’s protocol. The cDNA was then synthesized from total RNA using the High-Capacity cDNA Reverse Transcription Kit (Applied Biosystems Japan Ltd., Tokyo, Japan). Quantitative real time PCR analysis of individual cDNA was performed with ABI Prism 7500 (Applied Biosystems Japan Ltd., Tokyo, Japan) using TaqMan Gene Expression Assays (Applied Biosystems Japan Ltd., Tokyo, Japan) and primers, which were chosen on the basis of rat or mouse nucleotide sequences in the GenBank database (Table 1). PCR cycling conditions were 50 °C for 2 min, 95 °C for 10 min, followed by 40 cycles of 95 °C for 15 s and 60 °C for 1 min. The relative mRNA expression was normalized by b-actin mRNA. All the test chemicals lowered mRNA expression of these proteins. Therefore, effects of these three test compounds on the development of mucosal ulcer, dysplastic crypts, and colonic neoplasms may be related to the expression that was modified by feeding with test chemicals.

**Table 1:** Primer sequences used for real-time PCR assays.

Gene symbol	Primers	
	Rat	Mouse
**NF-** **κ** **B**	Forward: 5'-ctggcagctcttctcaaagc-3'	Mm00476361_m1 *
	Reverse: 5'-ccaggtcatagagaggctcaa-3'	
**Tnf-** **α**	Forward: 5'-cgagatgtggaactggcaga-3'	Mm00443258_m1 *
	Reverse: 5'-ctacgggcttgtcactcga-3'	
**IL-1β**	Rn00 580432_m1 *	Mm00434228_m1 *
**Stat3**	Forward: 5'-ttgtgatgcctccttgattgtc-3'	Mm00456961_m1 *
	Reverse: 5'-atcggaggcttagtgaagaagttc-3'	
**Hif-1** **α**	Rn00577560_ml *	Forward: 5'-cctggaaacgagtgaaagga-3'Reverse: 5'-tggtcagctgtggtaatcca-3'
** 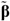 actin**	Forward: 5'-tcaggtcatcactatcggcaat-3'	Mm00607939_s1*
	Reverse: 5'-aaagaaagggtgtaaaacgca-3'	

* TaqMan (Assay ID#).

**Figure 14:**
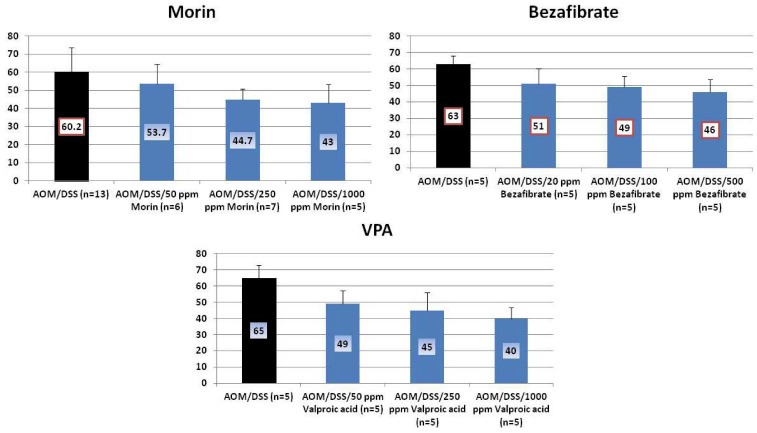
Immunohistochemical positivity (%) against survivin in colonic ADC cells that developed in mice or rats in the morin, bezafibrate, and VPA studies.

**Figure 15:**
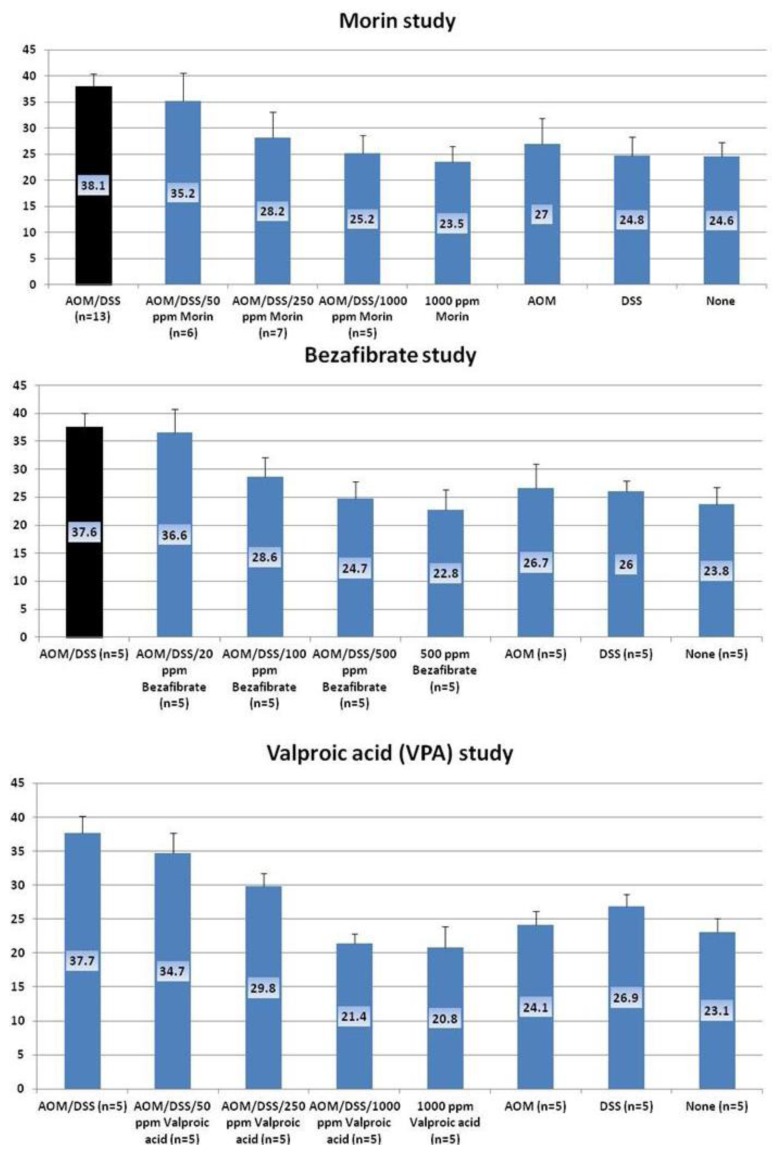
Polyamine content (nmol/mg) in the colonic mucosa of mice or rats in the morin, bezafibrate, and VPA studies.

Treatments with morin, bezafibrate, and VPA also lowered immunohistochemical positivity of survivin (Figure 14) in ADCs and polyamine contents (Figure 15) of colorectal mucosa. These effects may be also responsible for modulatory effects of these three compounds on colonic tumor development. However, further studies including dose selection and toxicity of the compounds should be conducted before going to clinical trials.

## 6. Conclusions

Chemoprevention is an important approach to decreasing cancer morbidity and mortality by the use of non-toxic natural or synthetic substances to reverse the processes of initiation, promotion, and subsequent progression of cancer. Evidence is rapidly accumulating that chronic inflammation contributes to carcinogenesis through increase of cell proliferation, angiogenesis, and metastasis in a number of neoplasms, including colorectal carcinoma. To investigate pathobiology of CRC developed in inflamed colorectum and search effective cancer chemopreventive agents against such colitis-related CRC, we developed mouse and rat models (TANAKA models) of inflammation-associated colorectal carcinogenesis. In this article, powerful tumor-promotion effect of inflammation induced by DSS in rodents that are initiated with a low dose of a colonic carcinogen is described. Also, our recent data on the modifying effects of morin, bezafibrate, and VPA on AOM/DSS-induced colorectal carcinogenesis is presented. I would stress that inflammatory stress and several cytokines produced by inflammatory cells are important in pathobiology of CRC development in colitic mucosa. These could be molecular targets for chemoprevention and/or therapy in CRC in IBD patients.
